# Unusual Metastatic Sites and Radioiodine Uptake in Patients of Differentiated Thyroid Carcinoma with Atypical Clinical Presentations: Utilization of ^131^ I-Whole-Body Scintigraphy with Regional SPECT/CT

**DOI:** 10.1055/s-0044-1779750

**Published:** 2024-04-01

**Authors:** Yeshwanth Edamadaka, Rahul V. Parghane, Sandip Basu

**Affiliations:** 1 Radiation Medicine Centre, Bhabha Atomic Research Centre, Tata Memorial Hospital Annexe, Jerbai Wadia Road, Parel, Mumbai, Maharashtra, India; 2 Radiation medicine center (BARC), Homi Bhabha National Institute, Mumbai, Maharashtra, India

**Keywords:** differentiated thyroid carcinoma, ^131^ I whole-body scintigraphy, SPECT/CT, urinary bladder metastasis, metacarpal metastasis

## Abstract

Differentiated thyroid carcinoma (DTC) usually is slow growing and carries a good prognosis. It most commonly tends to spread locally to regional lymph nodes in 20 to 60% of patients. The presence of distant metastasis impacts overall survival and prognosis. The lungs, bones, and the brain are typically involved in distant sites with less common metastatic sites that include the liver, kidney, skeletal muscle, adrenal glands, bladder, and skin. These unusual sites are rare and pose a diagnostic challenge and impact clinical decision-making to a great extent. The radioiodine ^131^ I whole-body scintigraphy with single-photon emission computed tomography/computed tomography can provide a thorough investigation of unusual sites of uptake leading to diagnosis of these metastases. We present a case series of DTC showing unusual sites of metastasis and/or radioiodine uptake in urinary bladder, in the third metacarpal bone of left hand and lastly in the forearm at postoperative hypertrophic scar area.

## Introduction

Thyroid cancers are the most common endocrine tumors, with differentiated thyroid carcinoma (DTC) accounting for more than 90% of cases. DTC are slow growing with good prognosis with nodal involvement in 20 to 60% of patients. 1 The presence of distant metastasis including the lungs, the bones, and the brain determines the prognosis with a reduction in overall survival. 2 Less common metastatic sites include the liver, kidney, skeletal muscle, adrenal glands, bladder, and skin. These uncommon sites pose a diagnostic challenge and impact clinical decision-making to a great extent. In such clinical situations, radioiodine whole-body scintigraphy (WBS) with single-photon emission computed tomography (SPECT)/computed tomography (CT) can lead to more insight into the diagnosis of rare metastasis. 3 4

Here we present a case series of three patients with DTC showing unusual sites of metastasis and/or radioiodine uptake in urinary bladder, in the third metacarpal bone of left hand and lastly in the forearm at postoperative skin.

## Case 1

**Fig. 1:**
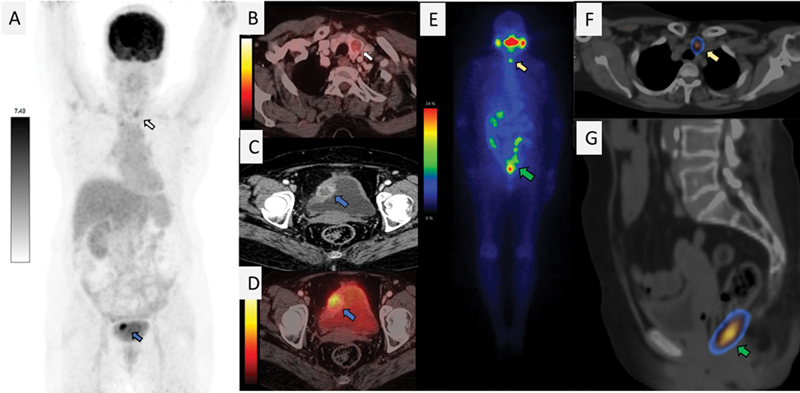
Case 1. [ ^18^ F] fluorodeoxyglucose positron emission tomography/computed tomography ^18^ F-FDG PET/CT maximum intensity projection image ( **A** ) showed intense focal uptake in bladder lesion ( *blue arrow* ) and low-grade uptake in left thyroid nodule ( *white arrow* ). Fused axial image ( **B** ) showed low-grade uptake in left thyroid nodule. Axial CT ( **C** ) and axial fused ( **D** ) images showed heterogeneously enhancing fluorodeoxyglucose avid bladder lesion. Post-therapy ^131^ I whole-body scintigraphy ( **E** ) showed single focus of tracer in neck ( *yellow arrow* ) and intense radioiodine concentration in pelvis in midline ( *green arrow* ). ^131^ I whole-body scintigraphy-single-photon emission computed tomography/computed tomography fused axial ( **F** ) image showed tracer localization to thyroid bed and fused sagittal ( **G** ) image showed linear physiological uptake in rectum, suggesting successful complete excision of urinary bladder metastatic tumor.

A 70-year-old female patient acutely presented with hematuria and was evaluated with abdomen and pelvis sonography that revealed polypoid growth measuring 2.8 × 1.9 cm in urinary bladder. Her cystoscopy showed a solitary sessile lesion in bladder. She underwent [ ^18^ F] fluorodeoxyglucose positron emission tomography/computed tomography imaging ( ^18^ F-FDG PET/CT), which demonstrated tracer avid lesion in urinary bladder and showed low-grade FDG avid hypodense lesion in left lobe of thyroid gland ( Fig. 1 ). The histopathological analysis of transurethral resection of bladder tumor specimen revealed a tumor cell arranged in a sinusoidal pattern and immunohistochemistry analysis showed the tumor cell was positive for Thyroid transcription factor-1 (TTF-1), paired box gene 8 (PAX-8), and thyroglobulin establishing a diagnosis for metastatic urinary bladder lesion of thyroid origin. She underwent total thyroidectomy with final diagnosis of DTC with urinary bladder metastasis and subsequently she was treated with high-dose radioiodine therapy (150 mCiof ^131^ I). The post-therapy ^131^ I-WBS showed tracer uptake in thyroid bed and focal tracer uptake in pelvic region. ^131^ I-SPECT/CT ( Fig. 1 ) confirmed pelvic tracer uptake to physiological uptake in rectum. This case was a rare site of metastasis in DTC with initial presentation of hematuria and incidentally detected thyroid nodule on ^18^ F-FDG PET/CT.

## Case 2

**Fig. 2:**
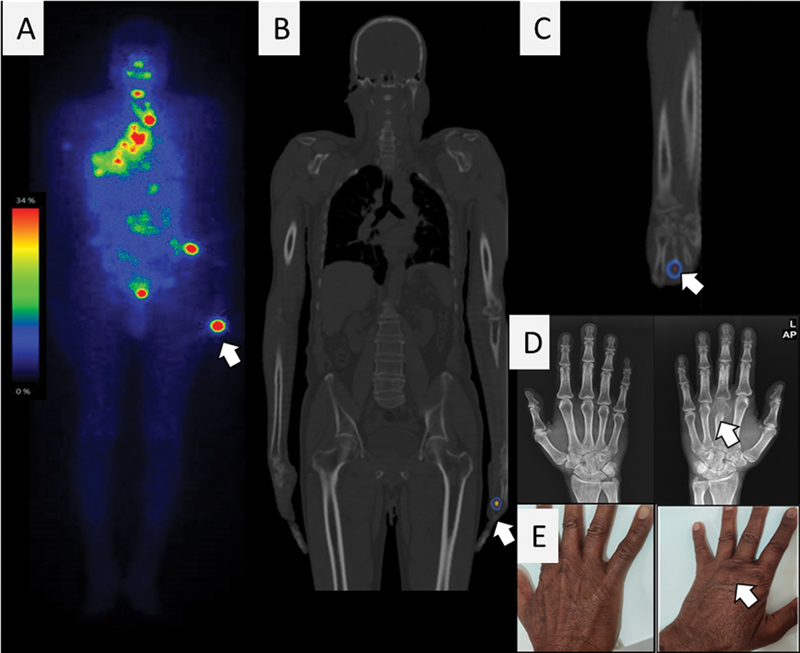
Case 2. Low-dose ^131^ I whole-body scintigraphy ( **A** ) showed multiple radioiodine concentration in thyroid bed, clavicle, mediastinal nodes, lung nodules, hand ( *white arrow* ), multiple pelvic lesions. ^131^ I whole-body scintigraphy-single-photon emission computed tomography/computed tomography fused coronal ( **B** ) and sagittal ( **C** ) images showed radioiodine concentration in hand localizing to metacarpal bone. X-ray ( **D** ) images of posteroanterior view of right and left hands showed expansile lytic lesion in third distal end of metacarpal bone in left side. Clinical image of patient hands ( **E** ) showed absent grooving on the dorsum of left hand with noticeable tender swelling.

A 68-year-old male patient noticed a swelling in neck moves with deglutition that was insidiously growing; ultrasound evaluation showed a solid cystic nodule in the left thyroid lobe and fine-needle aspiration cytology (FNAC) revealed follicular neoplasm. The patient underwent total thyroidectomy and histopathology showed DTC. Low-dose ^131^ I WBS showed tracer avidity ( Fig. 2 ) at neck, thoracic, abdominopelvis, and left-hand regions. On SPECT/CT, tracer uptake in left hand was localized to bony lesion of left third metacarpal bone. Upon enquiry, the patient revealed that he had the swelling and pain in left hand that led to reduced function much before the onset of thyroid swelling. The hand X-ray revealed an expansile lytic lesion with soft tissue component in third distal metacarpal bone on left side suggestive of metacarpal metastasis. On clinical examination, there was noticeable swelling on the dorsum of left hand and on palpation it was tender ( Fig. 2 ). This case was a rare site of metastasis in DTC detected on ^131^ I-WBS with SPECT/CT.

## Case 3

**Fig. 3:**
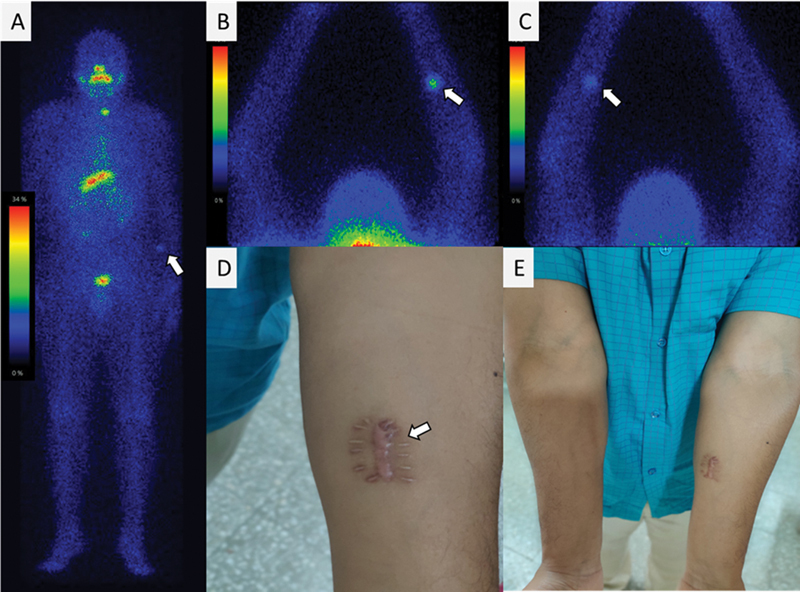
Case 3 **.** Post-therapy ^131^ I whole-body scintigraphy ( **A** ) showed single focal radioiodine concentration in neck and faint uptake in left forearm. Static high count images of ^131^ I with anterior ( **B** ) and posterior acquisition ( **C** ) showed the uptake in left forearm at surgical site of parathyroid implantation. Clinical image ( **D** , **E** ) showed patient forearms showed a hypertrophic scar which was nontender on palpation.

A 34-year-old male patient presenting with right neck swelling and an FNAC-proven DTC underwent total thyroidectomy along with bilateral neck dissection. The histopathological analysis was suggestive of differentiated papillary thyroid carcinoma, classical/conventional type in the right thyroid lobe with lymph node metastasis. Subsequently, patient was treated with high dose of ^131^ I radioiodine therapy, the post-therapy ^131^ I-WBS ( Fig. 3 ) demonstrating tracer uptake in thyroid bed and an additional focal tracer uptake in the left forearm. The patient had a history of surgically implanted parathyroid tissue in the left forearm during total thyroidectomy. On clinical examination, we noticed raised swelling with hypertrophic scar formation and without tenderness on palpation. This case was a rare site of radioiodine uptake on ^131^ I-WBS.

## Discussion

DTC has a better prognosis and longer survival, but in patients with metastatic disease, survival decreased and the prognosis became unfavorable. 5 A systematic review of rare metastasis in DTC showed brain (44%), skin (17%), and liver (8%) involvement as rare site of metastases. 6 Farina et al reported unusual sites of metastasis in 6 patients (0.4%) among 1,405 DTC patients studied. They reported three patients among six had cutaneous metastasis. The other three cases involved the kidney, adrenal, and liver, respectively. 7 Metastasis to the urinary bladder in DTC is extremely rare, with only a few cases reported in the literature. 8 9 10 11 12 The majority of these cases presented with complaints of hematuria, which led to the identification of lesions in the urinary bladder. Similar to this, our case 1 presented with hematuria that led to further investigation and incidental detection of thyroid nodule on ^18^ F-FDG-PET/CT. Solitary urinary bladder metastasis from DTC was eventually diagnosed proven on histopathology. Hence, the clinical suspicion of urinary bladder metastases should be raised in the presence of hematuria in DTC patients and it needs to be investigated further to determine the definitive clinical diagnosis.

The involvement of the hand in metastatic disease is uncommon and typically results from lung, breast, and kidney cancers. 13 14 The diagnosis of metastatic disease of the hand can be challenging since it may mimic an inflammatory, infectious, or metabolic disorder, with similar manifestations of inflammation, erythema, and pain. 15 16 In a similar manner, our second patient exhibited symptoms of hand swelling and pain, and subsequent imaging with SPECT/CT revealed the presence of metacarpal bone metastasis in DTC. We did not objectively verify the findings with tissue diagnosis, as case 2 presented with other site extensive skeletal metastasis and ^131^ I-WBS scan showed radioiodine concentration in left-hand lesion with anatomical imaging (SPECT/CT and X-ray) showed lytic lesion in third distal metacarpal bone, hence these imaging findings were highly suggestive of skeletal metastasis. Therefore, an invasive procedure was not suggested and tissue diagnosis not performed.

Skin metastasis originating from DTC is a rare occurrence with most of the cases reported in the head and neck region. The scalp is the most common site for skin metastasis. The rich network of blood vessels in the scalp, face, and chest can trap tumor emboli leading to metastatic foci. Nevertheless, the exact mechanism is unknown. 17 18 Some reports suggest the possibility of skin nodules being a result of needle tract implantation following FNAC procedures. 19 20 21 Kim et al 22 reported a case report of port site recurrence due to accidental spillage that was evident as an unusual pattern of radioiodine uptake in post-therapy on ^131^ I-WBS. The authors did not objectively verify these areas using cytological analysis, but on 7 years of follow-up, they reported no evidence of recurrence. Certain clinical and pathological findings are more indicative of implantation than traditional metastasis. These include recurrence at the site, a linear arrangement in skin and/or muscular seeding, and implantation occurring away from the surgical site. Similarly, our case 3 exhibited an unusual radioiodine uptake on ^131^ I-WBS with thyroglobulin levels 0.17 ng/mL at the surgically implanted site in the forearm, along with the formation of a hypertrophic scar, which may be indicative of implantation of thyroid tissue along with parathyroid. However, confirmatory tissue diagnosis would be considered in follow-up if any signs of recurrence noticed on clinical examination, biochemical, and imaging studies.

In case 1, ^131^ I-WBS and SPECT/CT helped in evaluating residual disease in urinary bladder bed region and it distinguished the physiological uptake in rectum, which resulted in deciding appropriate therapeutic radioiodine administration.

In case 2, SPECT/CT detected unusual site of flat bone metastasis that would imply requirement of multiple radioiodine therapies. Therefore, SPECT/CT demonstrated the benefit of deciding therapeutic dose and treatment management of both the above cases.

## Conclusion

The metastatic spread of DTC to uncommon sites such as the urinary bladder, hand, or skin remains a rare occurrence, but can substantially alter the clinical course if present. It is essential to identify these atypical presentations to ensure early diagnosis and prompt treatment. ^131^ I-WBS with SPECT/CT constitutes an important imaging modality for detecting these types of metastases in patients with DTC. Physicians must maintain a greater degree of awareness when DTC patients present with unusual symptoms or show unusual tracer uptake on ^131^ I-WBS to identify or rule out unusual metastases in DTC.
